# Physical and mental functioning trajectory classes among older adults and their association with specialized healthcare use

**DOI:** 10.1186/s12877-023-04157-w

**Published:** 2023-07-21

**Authors:** Jenni N. Ikonen, Timo Törmäkangas, Mikaela B. von Bonsdorff, Tuija M. Mikkola, Johan G. Eriksson, Markus J. Haapanen

**Affiliations:** 1 Department of General Practice and Primary Health Care, University of Helsinki, Helsinki, Finland; 2 Folkhälsan Research Center, Helsinki, Finland; 3 Population Health Unit, Finnish Institute for Health and Welfare, Helsinki, Finland; 4 Gerontology Research Center and Faculty of Sport and Health Sciences, University of Jyväskylä, Jyväskylä, Finland; 5 Clinicum, Faculty of Medicine, University of Helsinki, Helsinki, Finland; 6 Department of Obstetrics and Gynecology and Human Translational Research Programme, Yong Loo Lin School of Medicine, National University of Singapore, Singapore, Singapore; 7 Agency for Science, Technology and Research (A*STAR), Singapore Institute for Clinical Sciences (SICS), Singapore, Singapore; 8 Department of Medical Epidemiology and Biostatistics, Karolinska Institutet, Stockholm, Sweden

**Keywords:** Growth mixture model, Hospital service, Health service

## Abstract

**Background:**

Sex-specific physical and mental functioning trajectory classification could offer a way of understanding the differences in healthcare use at older age.

**Methods:**

Using latent growth mixture models, sex-specific physical and mental functioning trajectory classes were formed for 1991 participants (mean age 61.5 years) of the Helsinki Birth Cohort Study. Physical and mental functioning were evaluated with the SF-36 survey conducted in clinical examinations in 2001–2004, 2011–2013, and 2017–2018. First and follow-up outpatient visits, emergency visits, and hospital days were extracted from a national register between the first clinical examination and the year 2017. We used regression models to examine the associations between healthcare use and trajectory classes.

**Results:**

Two physical and mental functioning trajectory classes, *high* and *intermediate*, were observed for both sexes. The *intermediate* physical functioning trajectory class was associated with higher utilization rates of all examined specialized healthcare services (fully-adjusted IRRs varying 1.36–1.58; 95% CI = 1.03–1.79, 95% CI = 1.21–2.05) compared to the *high* trajectory class. Relative to the *high* trajectory class, the *intermediate* mental trajectory class was associated with the use of first outpatient visits (fully-adjusted IRRs 1.17, 95% CI = 1.03–1.33 for men, and 1.16, 95% CI = 1.04–1.30 for women). The findings were similar among both sexes.

**Conclusions:**

Compared to the *high* trajectory class, the *intermediate* physical functioning trajectory class was associated with greater specialized healthcare use and the *intermediate* mental trajectory class with first outpatient visits. Public health interventions should be considered to support functioning with aging.

**Supplementary Information:**

The online version contains supplementary material available at 10.1186/s12877-023-04157-w.

## Introduction

Globally, healthcare systems are facing challenges due to the increasing demands of rapidly aging populations. As most healthcare expenses in high-income countries result from specialized healthcare services provided mainly at hospitals [1], there is a need to understand factors associated with healthcare service use among older adults.

Healthy aging requires physical functioning that enables wellbeing at older age [2]. Decline in physical functioning, however, is inevitable at older age. Yet the rate of decline varies depending on environmental, sociodemographic, and lifestyle factors [2]. Decline in physical functioning might also be temporal or permanent making the declining process dynamic [2–4]. Therefore, distinguishable long-term patterns, trajectories, can be recognised in a population [2–4] and might be more reliable to understand declining physical functioning at older age. Moreover, although functional impairments have been associated with adverse health outcomes like mortality [5], and acute and long-term care use [6, 7], little is known on the association between physical functioning trajectory classes and specialized healthcare use [8, 9]. Further, since older women are reported to have poorer physical functioning than older men [10, 11], sex-specific physical functioning trajectory classification might offer valuable information on the possible sex differences in healthcare use.

In addition to changes in physical functioning, possible changes in social circles and chronic diseases at an older age may increase the likelihood of deterioration of mental health [12]. However, although the literature shows a higher likelihood of worse mental health among women compared to men [13, 14], no studies have examined the association between sex-specific mental functioning trajectory classes and healthcare use.

The aim of the current study is to examine the association between physical and mental functioning trajectory classes and specialized healthcare use among older men and women. We hypothesized that worse physical and mental functioning classes would be associated with greater specialized healthcare use compared to those with more stable classes.

## Methods

### Study population

The participants of the present study are a subsample of the Helsinki Birth Cohort Study of 8760 individuals born in Helsinki University Hospital between 1934 and 1944 [15]. A random sample of 2003 individuals participated in clinical examinations between the years 2001 and 2004. Follow-up examinations were performed between the years 2011 and 2013 and 2017 and 2018 as seen in the flowchart in Supplementary Fig. 1. Physical and mental functioning were assessed with the validated 36-Item Short Form Health Survey [16, 17] at all three study visits for those who participated (Supplementary Figure 1). Specialized healthcare use was obtained from a national specialized healthcare register starting from the first clinical examination date and ending at the end of the year 2017. Overall, 1999 participants had data on physical and mental functioning from at least one clinical visit to determine their physical and mental functioning. Eight individuals were excluded due to death in less than a year after the first clinical examination. The final study population consisted of 1991 individuals. All study participants gave a written informed consent before participating in any clinical procedures. The clinical study was performed in accordance with the Declaration of Helsinki and approved by the Coordinating Ethics Committee of the Hospital District of Helsinki and Uusimaa (THL/101/6.02.00/2019).

### Physical and mental functioning

Physical and mental functioning were assessed with the Finnish validated version of the 36-Item Short Form Health Survey Version 1.0 (SF-36), which measures self-reported general health and wellbeing [17]. The SF-36 consists of eight sub-domains which each include 2–10 items. The questionnaire has shown to be valid and practical instrument for older community dwelling older adults [18], and unidimensionality and reliability of the sub-scales have been proved among older adults [19]. The sub-domains are fundamentally associated with healthy functioning and are named as physical functioning (10 items), role limitation due to physical problems (4 items), bodily pain (2 items), general health perception (5 items), vitality (4 items), social functioning (2 items), role limitation due to emotional problems (3 items), and mental health (5 items). Each item was coded to range from 0 to 100 with higher scores indicating better functioning or well-being. Sub-domain scores were formed by calculating means of the items, and coded missing if less than half of the items were available. The scores of the sub-domains were then standardized [20] using the means and standard deviations of a US reference population from 1990, and then weighted using factor score coefficients from the US population. Next, the sub-domain scores were aggregated to create physical and mental component scores. Finally, both component scores were standardized using mean of 50 and standard deviation of 10 [21]. The same research assistant nurses carried out the procedures at all three clinical examinations. The functioning scores were further divided by 10 for analysis in mixture models.

### Healthcare utilization data

Healthcare utilization data were retrieved from the Care Register of Health Care, a national specialized healthcare register maintained by the Finnish Institute for Health and Welfare [22]. The register includes data on outpatient and inpatient healthcare visits from all hospitals in Finland. The data were retrieved starting from the first clinical examination date organized during 2001–2004 and continued until the end of the year 2017 or time of death where applicable. Emergency visits and first and follow-up outpatient visits were directly extracted from the register whereas hospital days were calculated by subtracting the admission date from the discharge date. If inpatient care exceeded 365 days, it was no longer treated as acute care and coded as 365 days in the dataset. Visits or hospital days were further summed for each study participant. First outpatient visits included all visits where a patient care relationship started with a new referral to a specific specialty while follow-up outpatient visits included all visits regarding the check-ups of the previously examined chronic diseases or injuries.

### Covariates

Covariates were assessed in the first clinical examination. Self-reported physician diagnosed chronic diseases or symptoms included myocardial infarction, angina pectoris, congestive heart failure, claudication, osteoporosis, stroke, depression, asthma, and emphysema. Self-reported chronic diseases were divided into three categories including ‘no diseases’, ‘one chronic disease’, and ‘two or more chronic diseases’. Frequency of smoking was assessed with a questionnaire and categorized into ‘never’, ‘former’, and ‘current smoker’. Self-reported alcohol consumption was categorized into ‘does not consume’, ‘two times a month at most’, and ‘three times a month or more’. Information on the participants’ highest educational attainment was obtained from Statistics Finland [23] in the year 2000 and divided into four groups: ‘basic or less or unknown’, ‘upper secondary’, ‘lower tertiary including polytechnic, vocational, and bachelor’s degree’, and ‘upper tertiary including master’s degree or higher’. Information on leisure time physical activity (LTPA) was assessed with the Kuopio Ischemic Heart Disease Risk Factor Study questionnaire [24]. Participants were asked about the duration, frequency, and intensity of LTPA during the past 12 months. Based on activity-specific metabolic equivalent (MET) values for each intensity grade, total leisure-time physical activity was calculated, and the results were expressed as METhours per week.

### Statistical analyses

To form physical and mental functioning trajectory classes, latent growth mixture models (LGMM) were fitted to the SF-36 summary component scores available at the clinical examinations in 2001–2004, 2011–2013 and 2017–2018. The analyses were performed separately for women and men with Mplus version 7 (1998–2015). Assuming non-response was missing at random, we used LGMM with Full Information Maximum Likelihood [25] to capture unobserved latent classes using all available data with similar physical and mental functioning trajectories, but which were relatively distinct across the latent classes over follow-up time. Within the latent classes, the component scores were used as outcomes in a growth model with time indexed through individually varying observation times corresponding to the chronological age of participants. Each latent class had their own growth parameters, intercept (indicating participant’s level of physical or mental functioning scores) and slope (indicating participant’s rate of linear changes in physical or mental functioning scores over time). For the classes, the average trajectory was based on the means of the intercept and slope parameters calculated at age means at each time point. Final classification of the participants was assigned by the highest group membership posterior probability which was based on data from each participant’s observed trajectory. The optimal number of latent classes was determined using the Bayesian Information Criterion (BIC), with lower values indicating a better fit of the model, and clarity of the participants’ classification. Clarity of classification into trajectory classes was assessed with percentage of individuals falling into latent classes based on membership posterior probabilities (high probabilities indicates clear separation of trajectory classes) and summarized by model entropy (an aggregate function of posterior probabilities), which ranges between zero and one, with higher values one indicating better class separation. Average trajectory classes were named based on the starting level and the slope of the graphical average trajectory: trajectory classes with better functioning between the numbers were named ‘high’ and the lower average trajectory classes ‘intermediate’ due to graphical presentation approximately in the middle of the chart. Trajectory classes with no or little slope were named ‘stable’ since a slight decrease in functioning was considered to be related to normal aging. Trajectories with more declining slopes were named ‘declining.

Means and standard deviations for continuous variables and proportions for categorical variables were calculated to compare baseline characteristics. Statistical differences in baseline characteristics were examined with the independent sample t-test, the Mann–Whitney U test, or the chi-square test. We used negative binomial regression models to examine the associations between physical and mental functioning trajectory classes and specialized healthcare use. Two models were created: Model 1 was adjusted for age, and Model 2 additionally for LTPA, education, number of chronic diseases, smoking, and alcohol consumption. The analyses were performed separately for men and women, and individual exposure times (offsets) were adjusted for the models. The results are shown as incidence rate ratios (IRRs). Significance level was set at 0.05, and p-values were not corrected for multiple testing. Further, negative binomial regression models were used to perform sensitivity analyses where we examined the associations between specialized healthcare use and physical and mental functioning trajectory classes with membership probability of 0.85 or higher. The analyses were performed in SPSS 26.0 for Windows (version 26.0, 1989–2020, SPSS Inc., Armonk, NY, USA).

## Results

### Physical and mental functioning trajectory classes

**Fig. 1:**
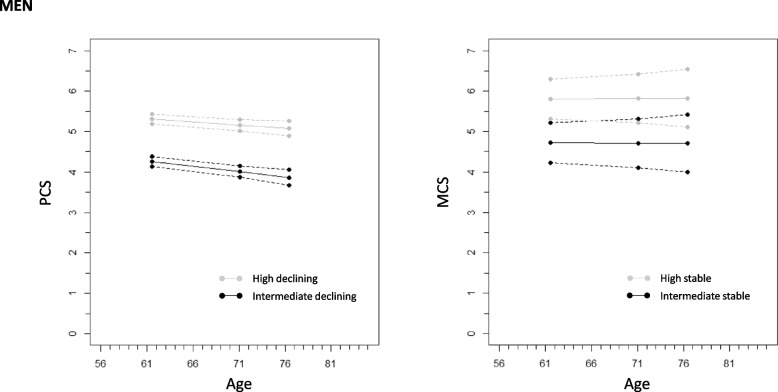
Model-expectation trajectories and 95% confidence intervals for physical (PCS) and mental (MCS) component score at means of individually varying age in three measurement time points from two groups (intermediate declining and high declining or intermediate stable or high stable) of growth mixture models among men. (Note: PCS and MCS were divided by 10 for the analyses resulting in the expected average trajectory scores seen in y-axis)

**Fig. 2:**
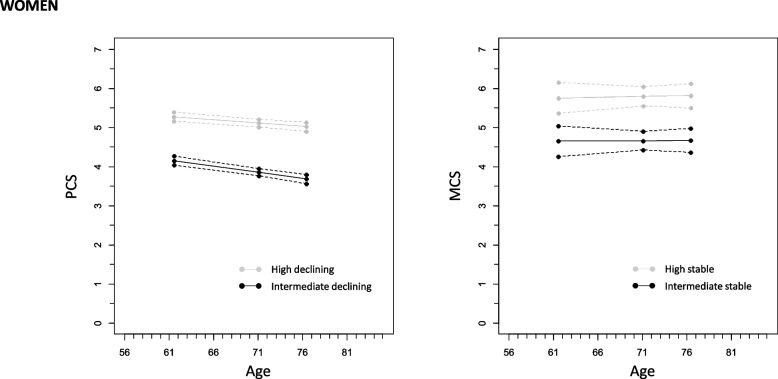
Model-expectation trajectories and 95% confidence intervals for physical (PCS) and mental (MCS) component score at means of individually varying age in three measurement time points from two groups (intermediate declining and high declining or intermediate stable or high stable) of growth mixture models among women. (Note: PCS and MCS were divided by 10 for the analyses resulting in the expected average trajectory scores seen in y-axis)

Figures 1 and 2 show physical and mental functioning trajectories in the two classes identified for men and women. The two physical functioning trajectory classes for an average participant among men and women were named *high declining* (65.6% of men, 55.5% of women) and *intermediate declining* (34.4 of men, 44.5% of women). As presented in Figs. 1 and 2, two mental functioning average trajectory classes were identified for both men and women: *high stable* (77.5% of men and 65.9% of women) and *intermediate stable* (22.5% of men and 34.1% of women). We refer to these trajectory classes as *high* and *intermediate* hereon in this manuscript. Supplementary Figs. 2 and 3 in the Supplement show all observed individual physical and mental functioning trajectories by sex.

### Characteristics of the study participants

Table 1 presents the characteristics of the study population. Men were more likely to have higher education, were more likely ex- or current smokers, were characterised by higher alcohol consumption, and had slightly higher annual utilization rates of emergency visits, first outpatient visits, and hospital days compared to women (Table 1). There were no differences between sexes in age, mean number of chronic diseases, and LTPA (Table 1). Supplementary Tables 1 and 2 in the Supplement show the characteristics of the study population according to the physical and mental functioning trajectory classes and sex.

**Table 1:** Baseline characteristics of the participants at the clinical examinations between the years 2001 and 2004

	All		Men		Women		*P*-value
	n	Mean (SD)	n	Mean (SD)	n	Mean (SD)	
Age	1991	61.5 (2.9)	921	61.5 (2.8)	1070	61.5 (3.0)	0.96
**Education**							< 0.001
Basic or less or unknown, %	709	35.6	273	29.6	436	40.7	
Upper secondary, %	511	25.7	225	24.4	286	26.7	
Lower level tertiary, %	528	87.8	272	29.5	256	23.9	
Upper level tertiary, %	243	12.2	151	16.4	92	8.6	
**Chronic diseases**							0.30
No diseases, %	1370	68.8	648	70.4	722	67.5	
1 chronic disease, %	430	21.6	185	20.1	245	22.9	
2 or more chronic diseases, %	191	9.6	88	9.6	103	9.6	
**Smoking**							< 0.001
Never, %	834	41.9	245	26.8	589	55.6	
Quitted earlier, %	669	33.6	419	45.8	250	23.6	
Current smoker, %	472	23.7	251	27.4	221	20.8	
**Alcohol use**							< 0.001
Does not use	146	7.3	69	7.5	77	7.2	
2 times/month at most	820	41.2	255	27.8	565	53.2	
3 times/month or more	1013	50.9	592	64.6	421	39.6	
**Physical activity (METhours/week)**	1955	46.0 (39.2)	900	45.2 (35.6)	1055	46.7 (41.1)	0.61
**Specialized healthcare use during the follow-up**							
Emergency visits/year	1991	0.29 (0.44)	921	0.35 (0.50)	1070	0.25 (0.39)	< 0.001
First outpatient visits/year	1991	0.36 (0.33)	921	0.37 (0.33)	1070	0.36 (0.34)	0.37
Follow-up outpatient visits/year	1991	1.9 (4.2)	921	2.2 (5.6)	1070	1.62 (2.6)	0.016
Hospital days/year	1991	2.5 (7.3)	921	3.1 (8.1)	1070	1.9 (6.5)	< 0.001

*SD* Standard deviation, *MET* Metabolic equivalent

### Physical and mental functioning trajectory classes and specialized healthcare use

Table 2 displays the results of regression analyses regarding physical functioning trajectory classes and specialized healthcare service use. Both men and women in the *intermediate* physical functioning trajectory class had higher utilization rates of any specialized healthcare service than those in the *high* trajectory class (Table 2). After all adjustments, the IRRs of both sexes were similar (Table 2).

**Table 2:** Incidence rate ratios (IRRs) for healthcare service use across physical functioning trajectory classes and sex

	Men		Women	
	Model 1^a^	Model 2^b^	Model 1^a^	Model 2^b^
	IRR (95% CI)	IRR (95% CI)	IRR (95% CI)	IRR (95% CI)
Emergency visit				
High declining	Ref	Ref	Ref	Ref
ntermediate declining	1.73 (1.47, 2.04)^***^	1.47 (1.25, 1.74)^***^	1.59 (1.35, 1.88)^***^	1.41 (1.18, 1.67)^***^
First outpatient visit				
High declining	Ref	Ref	Ref	Ref
Intermediate declining	1.52 (1.36, 1.70)^***^	1.43 (1.28, 1.60)^***^	1.70 (1.54, 1.88)^***^	1.56 (1.41, 1.73)^***^
Follow-up outpatient visit				
High declining	Ref	Ref	Ref	Ref
Intermediate declining	1.68 (1.21, 2.31)^**^	1.58 (1.21, 2.05)^***^	1.61 (1.36, 1.92)^***^	1.43 (1.20, 1.70)^***^
Hospital days				
High declining	Ref	Ref	Ref	Ref
Intermediate declining	1.76 (1.32, 2.35)^***^	1.38 (1.02, 1.87)^*^	1.70 (1.20, 2.40)^**^	1.36 (1.03, 1.79)^*^

*CI* Confidence interval

^*^ = *p* < 0.05, ^**^ = *p* < 0.01, ^***^ = *p* < 0.001

^a^Adjusted for age, *n* = 921 men and *n* = 1070 women

^b^ Adjusted for age, physical activity, education, number of chronic diseases, smoking, and alcohol consumption, *n* = 894 men and *n* = 1043 women

Table 3 shows the IRRs for specialized healthcare service use by mental functioning trajectory classes. Men and women classified into the *intermediate* trajectory class utilized first outpatient visits at higher rates in comparison to the *high* trajectory class. The utilization rates of first outpatient visits in the *intermediate* trajectory class were similar among both sexes (Table 3). In fully-adjusted models, no other statistically significant associations were observed (Table 3). Overall, the IRRs for specialized healthcare service use by mental functioning trajectory classes were smaller compared to the IRRs of physical functioning trajectory classes. The results were further confirmed in sensitivity analyses as seen in Supplementary Tables 3 and 4 in the Supplement.

**Table 3:** Incidence rate ratios (IRRs) for healthcare service use across mental functioning trajectory classes and sex

	Men			Women	
	Model 1^a^	Model 2^b^		Model 1^a^	Model 2^b^
	IRR (95% CI)	IRR (95% CI)		IRR (95% CI)	IRR (95% CI)
Emergency visit					
High declining	Ref	Ref		Ref	Ref
Intermediate declining	1.25 (1.03, 1.53)^*^	1.07 (0.90, 1.27)		1.21 (1.02, 1.44)^*^	1.08 (0.92, 1.28)
First outpatient visit					
High declining	Ref	Ref		Ref	Ref
Intermediate declining	1.21 (1.07, 1.38)^**^	1.17 (1.03, 1.33)^*^		1.24 (1.11, 1.39)^***^	1.16 (1.04, 1.30)^**^
Follow-up outpatient visit					
High declining	Ref	Ref		Ref	Ref
Intermediate declining	1.08 (0.84, 1.39)	1.10 (0.88, 1.37)		1.22 (1.02, 1.48)^*^	1.08 (0.90, 1.31)
Hospital days					
High declining	Ref	Ref		Ref	Ref
Intermediate declining	1.58 (1.14, 2.18)^**^	1.32 (0.92, 1.91)		1.09 (0.79, 1.52)	0.96 (0.72, 1.28)

*CI* Confidence interval

^*^ = *p* < 0.05, ^**^ = *p* < 0.01, ^***^ = *p* < 0.001

^a^Adjusted for age, *n* = 921 men and *n* = 1070 women

^b^Adjusted for age, physical activity, education, number of chronic diseases, smoking, and alcohol consumption, *n* = 894 men and *n* = 1043 women

## Discussion

In the present study, men and women in the *intermediate* physical functioning trajectory class had higher utilization rates of any specialized healthcare service compared with high functioning. Relative to the *high* mental trajectory class, the *intermediate* mental functioning trajectory class was associated with higher utilization rate of first outpatient visits. The findings were similar among both sexes.

We identified two physical and mental functioning trajectory classes for men and women. More than half of both men (65.5%) and women (55.5%) were included in the *high* physical functioning trajectory class suggesting relatively good physical functioning. In contrast, approximately one third of men (34.4%) and almost half of women (45.5%) belonged to the *intermediate* physical functioning trajectory class. Therefore, although decline over age was observed in both high and intermediate trajectory classes, a substantial proportion of the study population had an overall lower physical functioning level at older age. Among men and women, the observed individual trajectories in the *intermediate* class were less precisely captured by the trajectory model due to large variation in trajectory shapes suggesting more, possibly health-related variation in functioning patterns.

Most men (77.5%) and most women (65.9%) belonged to the *high* mental trajectory classes. In contrast, the *intermediate* mental functioning trajectory class was presented by approximately one fifth of men (22.5%) and one third of women (34.1%). Although specific interpretations cannot be done based on the proportions, poorer physical performance [10, 11] and mental health [13, 14] have been reported among older women compared to men.

The *intermediate* physical functioning trajectory class was consistently associated with specialized healthcare use among both sexes relative to the *high* physical functioning trajectory class. As far as we are aware, no other studies have examined the association between specialized healthcare use and physical functioning trajectory classes assessed as a function of the participants’ chronological age and separately for both sexes. However, other studies using latent growth mixture models have found associations between trajectory classes and disability [8, 9]. A Chinese study reported an association between late-onset disability trajectory classes and inpatient and outpatient care [8]. Further, late-onset and progressive disability trajectory classes were associated with expenses of inpatient and outpatient care [8]. Another study found progressive disability trajectory classification to have direct effects on healthcare service use including long-term care, inpatient care, and emergency services, and mediating effects from age and education to the use of same healthcare services among Taiwanese older adults [9]. Moreover, our results can be considered consistent with several previous studies which have concluded disability [6, 26, 27] and poor physical functioning [28] to increase the utilization of healthcare services. Some evidence suggests for a two-way association between physical functioning and chronic diseases. Chronic diseases are a known risk factor for physical functioning [29]. Therefore, in addition to age, chronic diseases may have contributed to decline in physical functioning trajectory and healthcare use. On the other hand, declining physical functioning might predispose individuals for chronic diseases [30, 31], possibly requiring hospital care. Possibly supporting the association between chronic diseases and physical functioning, we found a higher prevalence of specific chronic diseases in the intermediate physical functioning class compared to the high trajectory class in Supplementary Table 1.

Further, we found quite uniformly elevated utilization rates of specialized healthcare services among participants in the intermediate physical functioning trajectory class. We examined four types of visits including emergency visits, first and follow-up outpatient visits, and hospital days, and found the estimated IRRs to vary between 1.36 and 1.58 among men and women in the intermediate physical functioning trajectory class. The lowest increases in utilization rates were found for hospital days. However, the differences in the rates across these four types of visits were negligible suggesting similar increases in specialized healthcare utilization in all four types of visits among those in intermediate physical functioning trajectory class. This aligns with our hypothesis of physical functioning being an important factor to consider when studying healthcare service use among older adults.

We are not aware of studies to have examined the association between mental functioning trajectory classes and specialized healthcare use among older adults. In comparison to the *high* trajectory class, we found the *intermediate* mental functioning trajectory class to be associated with higher utilization rate of first outpatient visits among both men and women. The utilization rate was similar among both sexes. Although the literature regarding this topic is limited, it suggests that mental disorders, especially depression which is one of the most common mental health problems among older adults [32], are associated with outpatient [33–35] and inpatient care [34–36]. However, although both sexes in the *intermediate* mental functioning trajectory class had higher prevalence of depression and scored higher in Beck’s Depression Inventory [37] compared to the *high* trajectory in Supplementary Table 2, we found no association between trajectory classification and inpatient care. It must be noted, however, that a lower level of mental functioning does not necessarily mean a mental disease. Thus, the results of previous studies regarding healthcare use and mental disorders may not be directly comparable to our study. Future studies are needed to examine the associations between mental health trajectory classification and specialized healthcare use.

Overall, our study highlights the importance of maintaining higher levels of functioning, which may also result in lower utilization rates of specialized healthcare services and, therefore, help maintaining the sustainability of specialized healthcare services while populations age.Although physiological processes in aging make decline in physical functioning inevitable, physical activity improves or slows the process of decline [38]. In general, physical activity is thought to change the trajectory of decline [38]. Therefore, exercise promotion programs to all older adults to support physical functioning may lead to cost-savings in healthcare. Additionally, lifestyle factors, like poor diet, obesity, and smoking, have been associated with poor physical functioning emphasizing the role of public health interventions in the promotion of healthy lifestyle [39, 40]. Since the differences between trajectory classes were observed since the beginning of the follow-up, the interventions should start early enough to prevent decline in physical functioning at older age. Further, good mental functioning in long-term might decrease the need for first outpatient visits while aging. Thus, supporting mental functioning at older age may be beneficial to both older adults and to the healthcare system. More research, however, is needed to understand the associations between mental functioning trajectory classes and healthcare use before further conclusions for public health interventions can be reliably done.

The strengths of the current study are well collected data from a unique birth cohort and reliable register data. The study has, however, some limitations. First, since the clinical examinations were based on voluntariness, those with poor physical functioning may not have participated due to health reasons. This may have affected the formation of the trajectory classes. Second, the latent class mixture model is data-driven method, and we cannot address causality between physical functioning and increased specialized healthcare use, so it might be possible that greater healthcare use is linked to factors associated with poorer physical functioning. Third, the results may not be entirely generalizable outside Nordic countries since the healthcare systems of other countries differ. Finally, we did not have healthcare expenses available for our study.

In conclusion, poorer physical functioning trajectory classification among older adults was associated with greater specialized healthcare use. The* intermediate* mental health trajectory class, in turn, was only associated with the higher use of first outpatient visits. The associations found in the current study were similar among both sexes. Public health interventions should be considered to support physical functioning with aging. However, more research, especially regarding mental health trajectories and specialized healthcare use, is needed.

## Supplementary information

**Additional file 1: Supplementary Table 1.** Baseline characteristics by physical functioning trajectory classes among men and women. **Supplementary Table 2.** Baseline characteristics by mental functioning trajectory classes among men and women. **Supplementary Table 3.** Incidence rate ratios (IRRs) for healthcare service use across sex and physical functioning trajectory classes with membership probability of 0.85 or higher. **Supplementary Table 4.** Incidence rate ratios (IRRs) for healthcare service use across sex and mental functioning trajectory classes with membership probability of 0.85 or higher. **Supplementary Fig. 1.** Selection of the study participants. **Supplementary Fig. 2.** Observed (gray line), model-expectation (black line) and average (blue line) model-based trajectories for physical component score (PCS) in the intermediate and high declining classes among men and women. **Supplementary Fig. 3.** Observed (gray line), model-expectation (black line) and average (blue line) model-based trajectories for mental component score (MCS) in the intermediate and high stable classes among men and women.

## Data Availability

The datasets generated and/or analyzed during the current study are available from the corresponding author on reasonable request.
