# Supplementary material for: Physical and mental functioning trajectory classes among older adults and their association with specialized healthcare use
Source: BMC Geriatr. 2023 Jul 21;23:448. doi: 10.1186/s12877-023-04157-w (PMC10360356; doi:10.1186/s12877-023-04157-w)
Supplement: Supplementary file 1 — Additional file 1: Supplementary Table 1. Baseline characteristics by physical functioning trajectory classes among men and women. Supplementary Table 2. Baseline characteristics by mental functioning trajectory classes among men and women. Supplementary Table 3. Incidence rate ratios (IRRs) for healthcare service use across sex and physical functioning trajectory classes with membership probability of 0.85 or higher. Supplementary Table 4. Incidence rate ratios (IRRs) for healthcare service use across sex and mental functioning trajectory classes with membership probability of 0.85 or higher. Supplementary Fig. 1. Selection of the study participants. Supplementary Fig. 2. Observed (gray line), model-expectation (black line) and average (blue line) model-based trajectories for physical component score (PCS) in the intermediate and high declining classes among men and women. Supplementary Fig. 3. Observed (gray line), model-expectation (black line) and average (blue line) model-based trajectories for mental component score (MCS) in the intermediate and high stable classes among men and women. [file 12877_2023_4157_MOESM1_ESM.pdf]

Physical and mental functioning trajectory classes among older adults and their association with specialized healthcare use

Supplementary Material

Supplementary Table 1. Baseline characteristics by physical functioning trajectory classes among men and women

Supplementary Table 2. Baseline characteristics by mental functioning trajectory classes among men and women

Supplementary Table 3. Incidence rate ratios (IRRs) for healthcare service use across sex and physical functioning trajectory classes with membership probability of 0.85 or higher.

Supplementary Table 4. Incidence rate ratios (IRRs) for healthcare service use across sex and mental functioning trajectory classes with membership probability of 0.85 or higher.

Supplementary Figure 1. Selection of the study participants.

Supplementary Figure 2. Observed (gray line), model-expectation (black line) and average (blue line) model-based trajectories for physical component score (PCS) in the intermediate and high declining classes among men and women.

Supplementary Figure 3. Observed (gray line), model-expectation (black line) and average (blue line) model-based trajectories for mental component score (MCS) in the intermediate and high stable classes among men and women.

Supplementary Table 1. Baseline characteristics by physical functioning trajectory classes among men and women.

	Men				Women			
	Intermediate physical functioning trajectory class		High physical functioning trajectory class		Intermediate physical functioning trajectory class		High physical functioning trajectory class	
	n	Mean (SD)	n	Mean (SD)	n	Mean (SD)	n	Mean (SD)
Age	317	61.7 (2.9)	604	61.5 (2.8)	476	62.0 (3.2)	594	61.2 (2.9)
Education								
Basic or less or unknown, %	120	37.9	153	25.3	213	44.7	223	37.5
Upper secondary, %	96	30.3	129	21.4	134	28.2	152	25.6
Lower level tertiary, %	77	24.3	195	32.3	100	21.0	156	26.3
Upper level tertiary, %	24	7.6	127	21.0	29	6.1	63	10.6
Chronic diseases								
No diseases	176	55.5	472	78.1	251	52.7	471	79.3
1 chronic disease	84	30.3	101	21.4	141	29.6	104	17.5
2 or more chronic diseases	57	18.0	31	5.1	84	17.6	19	3.2
Diseases								
Cardiovascular disease, %	177	56.0	209	34.7	232	48.8	174	29.4
Diabetes, %	51	16.1	33	5.5	45	9.5	18	3.0
Asthma/COPD, %	54	17.1	45	7.5	94	19.8	52	8.8
Depression, %	48	15.2	47	7.1	100	21.1	53	9.0
Smoking								
Never, %	67	21.3	178	29.7	264	55.8	325	55.4
Quitted earlier, %	141	44.8	278	46.3	107	22.6	143	24.4
Current smoker, %	107	34.0	144	24.0	102	21.6	119	20.3
Alcohol use								
Does not use, %	26	8.3	43	7.2	43	9.1	34	5.8
2 times/month at most, %	100	31.7	155	25.8	263	55.7	302	51.1
3 times/month or more	189	60.0	403	67.1	166	35.2	255	43.1
Physical activity (MET hours/week)	303	42.7 (36.0)	597	46.5 (37.2)	466	47.3 (47.4)	589	46.3 (35.4)
Self-reported physical condition								
Very good	2	0.6	43	7.1	5	1.1	34	5.7
Fairly good	47	14.8	271	44.9	89	18.7	270	45.5
Satisfactory	140	44.2	239	39.6	205	43.1	232	39.1
Quite poor	113	35.8	48	7.9	149	31.3	55	9.3
Very poor	15	4.7	3	0.5	28	5.9	3	0.5
Grip strength	314	38.8 (9.5)	603	41.0 (9.4)	415	21.9 (6.4)	534	23.7 (6.1)
BDI	315	7.8 (5.9)	603	4.2 (4.2)	460	8.8 (6.4)	586	5.5 (5.3)

SD = standard deviation; MET = metabolic equivalent; BDI = Beck's Depression Inventory

Supplementary Table 2. Baseline characteristics by mental functioning trajectory classes among men and women

	Men				Women			
	Intermediate mental functioning trajectory class		High mental functioning trajectory class		Intermediate mental functioning trajectory class		High mental functioning trajectory class	
	n	Mean (SD)	n	Mean (SD)	n	Mean (SD)	n	Mean (SD)
Age	207	61.8 (2.7)	714	61.5 (2.8)	365	61.9 (3.1)	705	61.3 (3.0)
Education								
Basic or less or unknown, %	68	32.9	205	28.7	154	42.2	282	40.0
Upper secondary, %	57	27.5	168	23.5	97	26.6	189	26.8
Lower level tertiary, %	49	23.7	223	31.2	89	24.4	167	23.7
Upper level tertiary, %	33	15.9	118	16.5	25	6.8	67	9.5
Chronic diseases								
No diseases	124	59.9	524	73.4	202	55.3	520	73.8
1 chronic disease	53	25.6	132	18.5	106	29.0	139	19.7
2 or more chronic diseases	30	14.5	58	8.1	57	15.6	46	6.5
Diseases								
Cardiovascular disease, %	101	49.3	285	39.9	145	39.9	261	37.1
Diabetes, %	24	11.7	60	8.4	23	6.3	40	5.7
Asthma/COPD, %	19	9.3	80	11.2	57	15.7	89	12.6
Depression, %	47	22.9	44	6.2	98	27.0	55	7.8
Smoking								
Never, %	50	24.5	195	27.4	202	55.8	387	55.4
Quitted earlier, %	86	42.2	333	46.8	83	22.9	167	23.9
Current smoker, %	68	33.3	183	25.7	77	21.3	144	20.6
Alcohol use								
Does not use, %	14	6.8	55	7.7	34	9.4	43	6.1
2 times/month at most, %	60	29.3	195	27.4	186	51.4	379	54.1
3 times/month or more	131	63.9	461	64.8	142	39.2	279	39.8
Physical activity (MET hours/week)	199	45.7 (38.2)	701	45.1 (36.5)	358	47.9 (43.0)	697	46.1 (40.1)
Self-reported physical condition								
Very good	8	3.9	37	5.2	8	2.2	31	4.4
Fairly good	45	21.7	273	38.2	98	26.8	261	37.0
Satisfactory	86	41.5	293	41.0	148	40.5	289	41.0
Quite poor	58	28.0	103	14.4	96	26.3	108	15.3
Very poor	10	4.8	8	1.1	15	4.1	16	2.3
Grip strength	206	39.2 (10.0)	711	40.5 (9.3)	330	22.1 (6.2)	619	23.4 (6.3)
BDI	207	9.6 (6.5)	711	4.2 (3.9)	360	10.8 (7.1)	686	5.0 (4.2)

SD = standard deviation; MET = metabolic equivalent; BDI = Beck's Depression Inventory

Supplementary Table 3. Incidence rate ratios (IRRs) for healthcare service use across sex and physical functioning trajectory classes with membership probability of 0.85 or higher.

	Men		Women	
	Model 1[a] IRR (95% CI)	Model 2[b] IRR (95% CI)	Model 1[a] IRR (95% CI)	Model 2[b] IRR (95% CI)
Emergency visit				
High declining	Ref.	Ref.	Ref.	Ref.
Intermediate declining	2.15 (1.77, 2.61)***	1.75 (1.42, 2.17)***	2.38 (1.97, 2.88)***	2.06 (1.69, 2.52)***
First outpatient visit				
High declining	Ref.	Ref.	Ref.	Ref.
Intermediate declining	1.80 (1.58, 2.05)***	1.67 (1.46, 1.92)***	2.07 (1.82, 2.36)***	1.90 (1.66, 2.17)***
Follow-up outpatient visit				
High declining	Ref.	Ref.	Ref.	Ref.
Intermediate declining	2.20 (1.61, 3.00)***	2.01 (1.52, 2.66)***	2.34 (1.87, 2.91)***	2.12 (1.69, 2.64)***
Hospital days				
High declining	Ref.	Ref.	Ref.	Ref.
Intermediate declining	2.37 (1.67, 3.36)***	1.94 (1.34, 2.80)***	3.85 (2.42, 6.10)***	2.86 (1.98, 4.12)***

[a] Adjusted for age, n = 669 men and n = 676 women

[b] Adjusted for age, physical activity, education, number of chronic diseases, smoking, and alcohol consumption, n = 649 men and n = 656 women

CI = confidence interval

*= p < 0.05, ** = p < 0.01, ***= p < 0.001

Supplementary Table 4. Incidence rate ratios (IRRs) for healthcare service use across sex and mental functioning trajectory classes with membership probability of 0.85 or higher.

	Men		Women	
	Model 1[a] IRR (95% CI)	Model 2[b] IRR (95% CI)	Model 1[a] IRR (95% CI)	Model 2[b] IRR (95% CI)
Emergency visit				
High declining	Ref.	Ref.	Ref.	Ref.
Intermediate declining	1.28 (1.03, 1.61)*	1.08 (0.89, 1.31)	1.26 (1.03, 1.55)*	1.09 (0.89, 1.32)
First outpatient visit				
High declining	Ref.	Ref.	Ref.	Ref.
Intermediate declining	1.23 (1.06, 1.42)**	1.17 (1.01, 1.35)*	1.29 (1.15, 1.46)***	1.20 (1.07, 1.36)**
Follow-up outpatient visit				
High declining	Ref.	Ref.	Ref.	Ref.
Intermediate declining	1.14 (0.85, 1.51)	1.15 (0.90, 1.46)	1.31 (1.05, 1.62)*	1.14 (0.91, 1.42)
Hospital days				
High declining	Ref.	Ref.	Ref.	Ref.
Intermediate declining	1.74 (1.20, 2.51)**	1.41 (0.92, 2.15)	1.34 (0.94, 1.93)	0.95 (0.69, 1.33)

[a] Adjusted for age, n = 767 men and n = 753 women

[b] Adjusted for age, physical activity, education, number of chronic diseases, smoking, and alcohol consumption, n = 745 men and n = 737 women

CI = confidence interval

*= p < 0.05, ** = p < 0.01, ***= p < 0.001

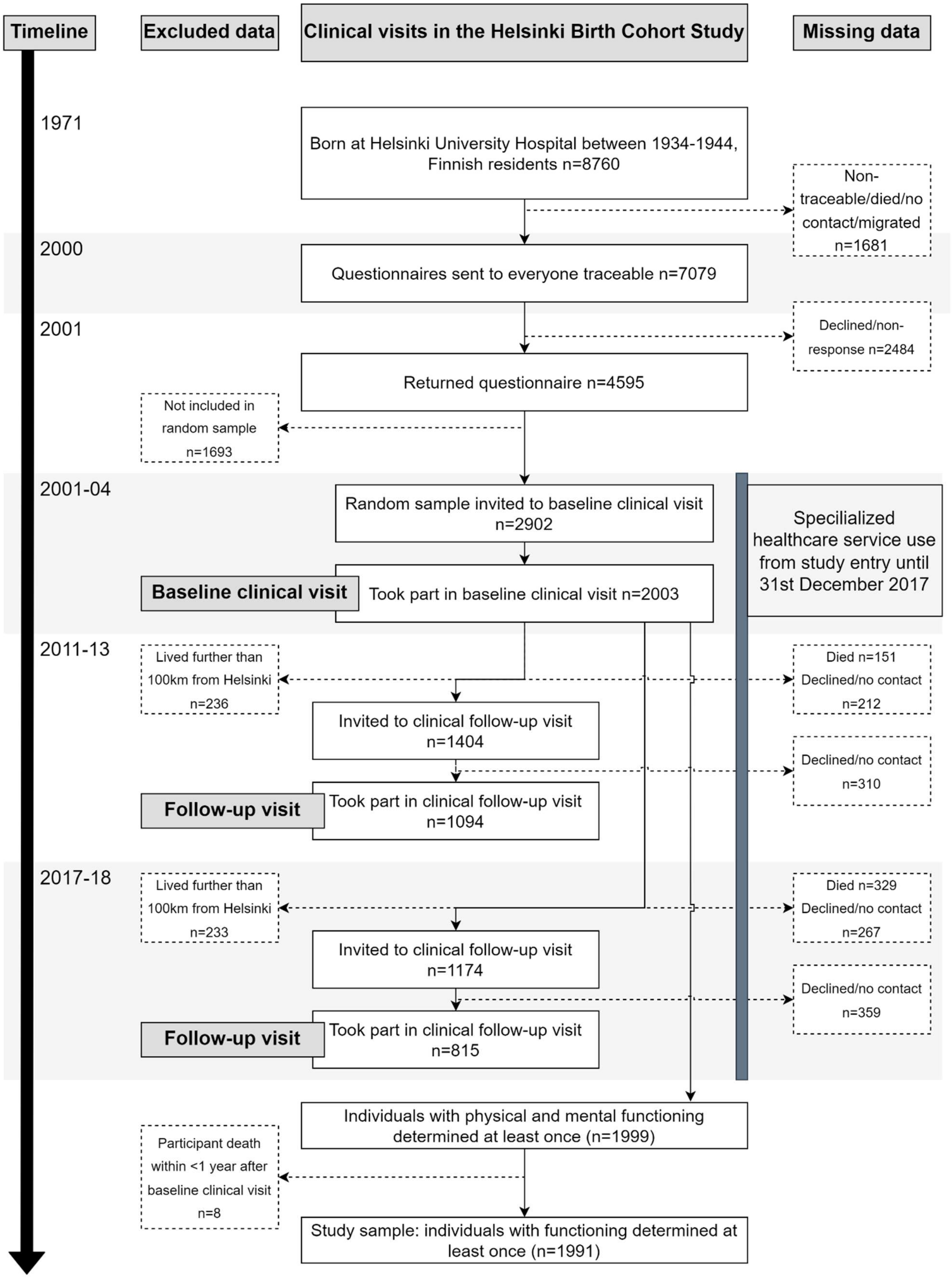

Supplementary Figure 1. Selection of the study participants.

Physical Component Score (PCS)

Men

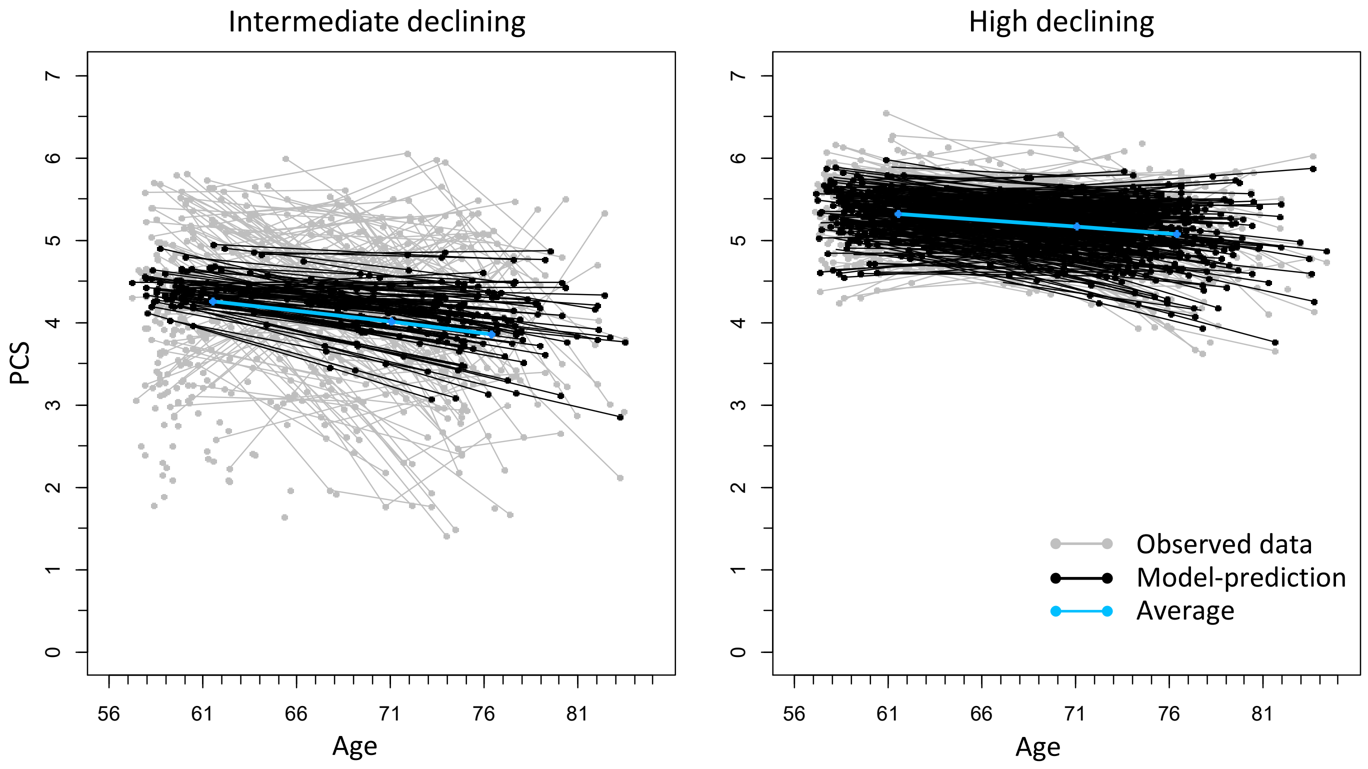

Women

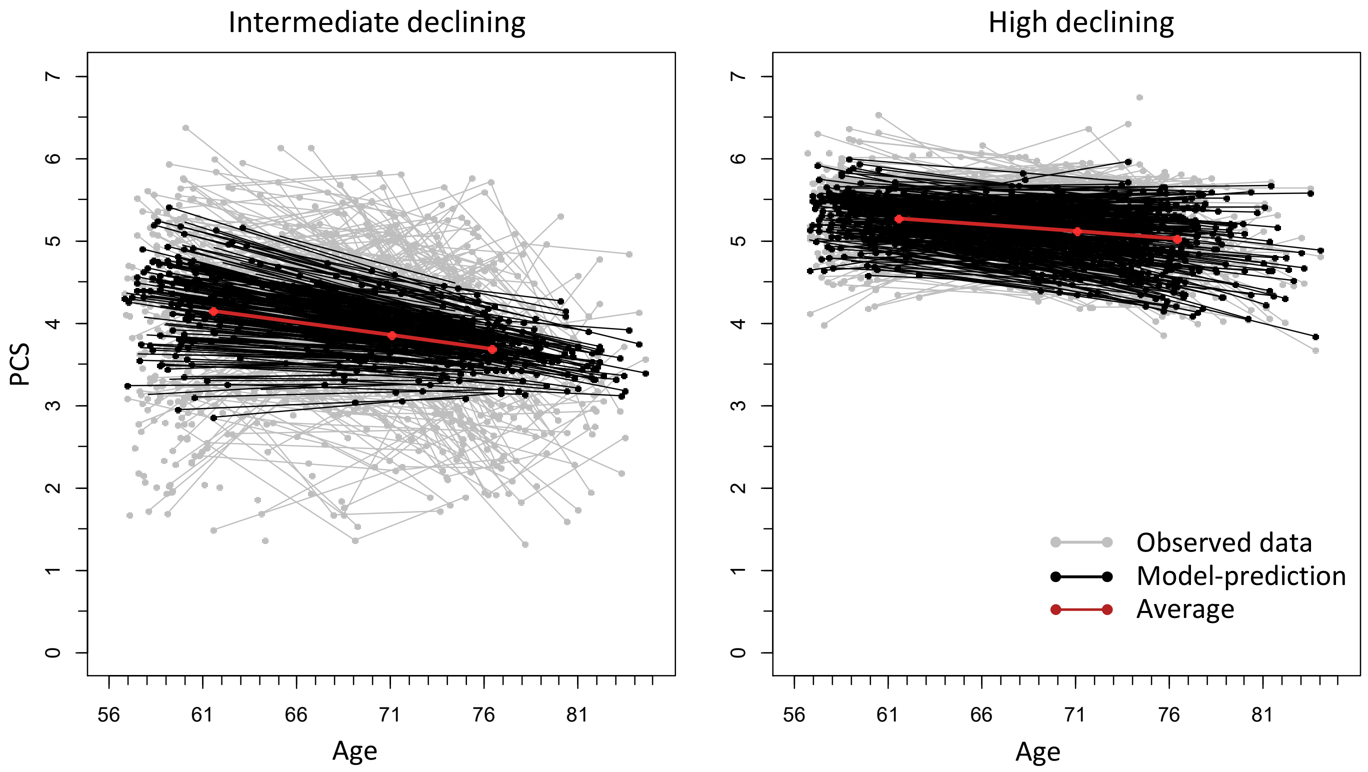

Supplementary Figure 2. Observed (gray line), model-expectation (black line) and average (blue line) model-based trajectories for physical component score (PCS) in the intermediate and high declining classes among men and women.

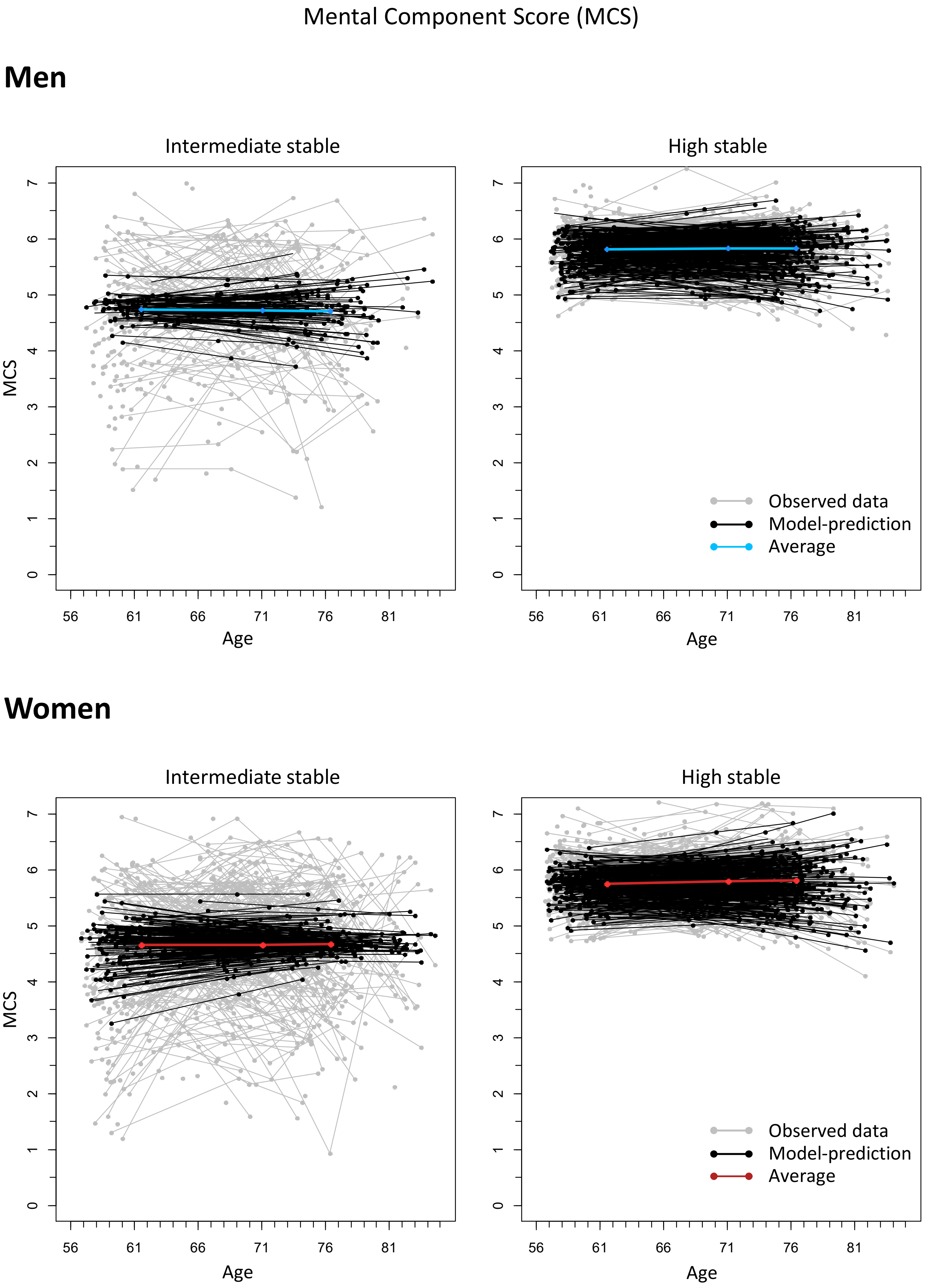

Supplementary Figure 3. Observed (gray line), model-expectation (black line) and average (blue line) model-based trajectories for mental component score (MCS) in the intermediate and high stable classes among men and women.
